# Peripheral blood but not synovial fluid natural killer T cells are biased towards a Th1-like phenotype in rheumatoid arthritis

**DOI:** 10.1186/ar1695

**Published:** 2005-02-18

**Authors:** Loes Linsen, Marielle Thewissen, Kurt Baeten, Veerle Somers, Piet Geusens, Jef Raus, Piet Stinissen

**Affiliations:** 1 Biomedisch Onderzoeksinstituut, Limburgs Universitair Centrum and School of Life Sciences, Transnationale Universiteit Limburg, Universitaire Campus, Diepenbeek, Belgium

## Abstract

Natural killer T (NKT) cells have been implicated in the regulatory immune mechanisms that control autoimmunity. However, their precise role in the pathogenesis of rheumatoid arthritis (RA) remains unclear. The frequency, cytokine profile and heterogeneity of NKT cells were studied in peripheral blood mononuclear cells (PBMCs) from 23 RA patients and 22 healthy control individuals, including paired PBMC–synovial fluid samples from seven and paired PBMC–synovial tissue samples from four RA patients. Flow cytometry revealed a decreased frequency of NKT cells in PBMCs from RA patients. NKT cells were present in paired synovial fluid and synovial tissue samples. Based on the reactivity of PBMC-derived NKT cells toward α-galactosylceramide, RA patients could be divided into responders (53.8%) and nonresponders (46.2%). However, NKT cells isolated from synovial fluid from both responders and nonresponders expanded upon stimulation with α-galactosylceramide. Analysis of the cytokine profile of CD4^+ ^and CD4^- ^PBMC derived NKT cell lines from RA patients revealed a significantly reduced number of IL-4 producing cells. In contrast, synovial fluid derived NKT cell lines exhibited a Th0-like phenotype, which was comparable to that in healthy control individuals. This suggests that synovial fluid NKT cells are functional, even in patients with nonresponding NKT cells in their blood. We conclude that, because the number of Vα24^+^Vβ11^+^CD3^+ ^NKT cells is decreased and the cytokine profile of blood-derived NKT cells is biased toward a Th1-like phenotype in RA patients, NKT cells might be functionally related to resistance or progression of RA. Providing a local boost to the regulatory potential of NKT cells might represent a useful candidate therapy for RA.

## Introduction

Natural killer T (NKT) cells are a distinct subset of lymphocytes that share the characteristics of both T cells and natural killer cells. They express a semi-invariant TCR (TCR Vα24Jα18 and Vβ11 in human; Vα14Jα281 and Vβ8, Vβ7 or Vβ2 in mouse) and recognize glycolipid antigens presented by the major histocompatibility complex class I-like molecule CD1d [1]. Two subsets can be distinguished [2,3]: CD4^+ ^NKT cells that produce T-helper (Th)1-type and Th2-type cytokines, and CD4^-^CD8^- ^(double negative) NKT cells that primarily produce Th1-type cytokines. The ability to secrete cytokines and chemokines rapidly is thought to underlie their regulatory function in a variety of diseases, including cancer and autoimmunity [4]. Although the natural ligand of NKT cells remains to be elucidated, it has been reported that the sponge derived glycolipid α-galactosylceramide (α-GalCer) is a potent activator of mouse and human NKT cells, both *in vitro *and *in vivo *[5,6]. When α-GalCer is administered to mice it polarizes the adaptive immune response toward production of Th2 cytokines [7,8], which therefore raises the possibility that α-GalCer can temper or even prevent Th1-mediated autoimmune diseases.

Several studies have shown that NKT cells are decreased or dysfunctional in autoimmune conditions such as insulin-dependent diabetes mellitus, systemic sclerosis, systemic lupus erythematosus, rheumatoid arthritis (RA) and multiple sclerosis [9-12]. Significant therapeutic effects of α-GalCer have been demonstrated in animal models of autoimmunity, such as experimental allergic encephalomyelitis [13-15] and nonobese diabetic mice [16,17].

Because the NKT/CD1d system is phylogenetically conserved among mammals, findings in mice are expected to have a direct parallel in humans. The NKT cell frequency in peripheral blood mononuclear cells (PBMCs) is lower in humans than in mice [1], which may be an obstacle in translating results from animal studies to the clinic. However, results from a phase I study conducted in advanced cancer patients revealed that treating patients with α-GalCer can increase NKT cell numbers above pretreatment levels. This again indicates that α-GalCer could be applied to the treatment of patients with autoimmune disease [18].

RA is an autoimmune disease that is characterized by a chronic inflammation of the joints, followed by progressive destruction of cartilage and underlying bone [19]. Autoreactive Th1 T cells are believed to play a major role in the disease process [20-22]. In RA patients, the frequency of NKT cells is decreased, but the functional characteristics of NKT cells have not yet been fully elucidated. Chiba and coworkers [23] demonstrated that administration of a truncated form of α-GalCer to mice suffering from collagen-induced arthritis – a frequently used animal model of RA – resulted in protection from disease, indicating that this might represent a therapy that can enhance NKT cell numbers in RA patients.

In the present study we analyzed the frequency, functional characteristics and heterogeneity of NKT cells in peripheral blood, synovial fluid and synovial tissue from RA patients. In parallel, we assessed these parameters in α-GalCer-stimulated short-term cell lines of both peripheral blood and synovial fluid NKT cells. We found that NKT cells were decreased and had altered functional properties in peripheral blood, but they were not impaired in synovial fluid from RA patients. Our data indicate that NKT cells may be involved in the disease process of RA and that a strategy to boost the regulatory potential of NKT cells might be useful in the treatment of RA.

## Materials and methods

### Patients and healthy control individuals

NKT cell characteristics were examined in 23 RA patients (mean age 52.1 ± 2.0 years, 11 males and 12 females, mean disease duration 8.0 ± 1.6 years), who were diagnosed in accordance with the criteria of the American College of Rheumatology [24], and in 22 healthy individuals (mean age 48.6 ± 2.0 years, 10 males and 12 females). When RA patients presented with a swollen knee, paired peripheral blood and synovial fluid samples were obtained. Synovial tissue samples were obtained from four RA patients after total knee/hip arthroplasty. Patients were informed about the purpose of the study and gave written consent. Approval for the study was granted by our ethics committee. Patient characteristics are summarized in Table 1.

### Flow cytometric analysis of natural killer T cells

Expression of cell surface markers was analyzed by flow cytometry. Fluorescein isothiocyanate (FITC)-labelled anti-TCR Vα24 and phycoerythrin (PE)-labelled TCR Vβ11 were purchased from Serotec Ltd (Oxford, UK). Anti-CD3-PE, anti-CD3-PerCP, anti-CD4-FITC, anti-CD8-PE, anti-CD25-FITC, anti-IFN-γ-FITC and anti-IL-4-PE were obtained from Becton Dickinson (Erembodegem, Belgium). The frequency of invariant NKT cells was estimated using three-colour anti-Vα24/anti-Vβ11/anti-CD3 staining. For intracellular cytokine detection, α-GalCer expanded Vα24^+^Vβ11^+ ^NKT cells or Vα24^+ ^isolated NKT cell lines were stimulated with 25 ng/ml phorbol-12-myristate-13-acetate and 1 μg/ml ionomycine in the presence of 10 μg/ml brefeldin A for 4 hours. Intracellular staining was performed as previously described [25]. Cells were analyzed on a FACSCalibur flow cytometer using Cellquest software (Becton Dickinson).

### Direct *ex vivo *analysis of the cytokine profile of natural killer T cells by ELISPOT

ELISPOT procedure was performed as previously described [25]. Briefly, 2 × 10^5 ^PBMCs were stimulated with 100 ng/ml α-GalCer in anti-IFN-γ or anti-IL-4 (Mabtech, Nacka, Sweden) coated nitrocellulose bottomed plates (Millipore Corp, Bedford, MA, USA). After 20 hours of culture, biotinylated anti-IFN-γ or anti-IL-4 antibody (Mabtech) was added for 2 hours followed by incubation with streptavidin-alkaline phosphatase (Mabtech) and NBT/BCIP (Nitro Blue Tetrazolium/5-Bromo-4 Chloro-3-Indolyphosphate; Pierce, Rockford, IL, USA) as substrate. The number of cytokine-secreting cells was calculated by subtracting the number of spots in control wells (without antigen) from the number of spots obtained in the presence of α-GalCer.

### Expansion and culture of Vα24^+^Vβ11^+ ^natural killer T cells

PBMCs and synovial fluid mononuclear cells (SFMCs) were isolated using Ficoll-Hypaque (Sigma Diagnostics, St Louis, MO, USA) density gradient centrifugation. PBMCs and SFMCs were cultured in the presence of 100 ng/ml α-GalCer (Kirin Brewery Ltd, Gunma, Japan) at a density of 7.5 × 10^5 ^cells/ml RPMI supplemented with 10% heat-inactivated foetal bovine serum, 1 mmol/l sodiumpyruvate and 1% nonessential amino acids (Invitrogen, Merelbeke, Belgium). After 7 days, cells were re-stimulated with irradiated autologous, α-GalCer pulsed PBMCs and supplemented with 2 U/ml recombinant human IL-2 (Roche Diagnostics, Brussels, Belgium). On day 7 after re-stimulation, NKT cells were isolated using Vα24^+ ^magnetic isolation (EasySep; Stemcell Technologies, Meylan, France), in accordance with the manufacturer's instructions. Reactivity of the isolated NKT cells toward α-GalCer was tested in a standard [^3^H]thymidine incorporation assay. During the last 16 hours of culture, cells were pulsed with 1 μCi [^3^H]thymidine (Amersham, Buckinghamshire, UK) and subsequently harvested using an automated cell harvester (Pharmacia, Uppsala, Sweden). Incorporated radioactivity was measured using a β-plate liquid scintillation counter (Wallac, Turku, Finland). A NKT cell line was considered to be antigen reactive when the mean counts per minute in the presence of α-GalCer exceeded 1000 and the stimulation index (mean counts with α-GalCer/mean counts without α-GalCer) was greater than 3.

### Analysis of clonal heterogeneity by T-cell receptor CDR3 region fragment length analysis

RNA was isolated from snap frozen synovial tissue samples using the Absolutely RNA RT-PCR Miniprep Kit (Stratagene, Amsterdam, The Netherlands). For isolation of total RNA from PBMCs, SFMCs and isolated NKT cells, the High Pure total RNA Isolation kit (Roche Diagnostics, Brussels, Belgium) was used, in accordance with the manufacturer's instructions. RNA was reverse transcribed into cDNA using AMV reverse transcriptase and an oligo-dT primer (Promega, Madison, WI, USA).

CDR3 spectratyping analysis was performed as described previously [26]. Briefly, 2 μl cDNA was used for first-round PCR analysis performed in 1 × PCR buffer, 0.9 U Taq polymerase, 0.02 mmol/l dNTP mix (all from Roche Diagnostics), 1 μmol/l forward primer specific for TCR Vα24 (5'-GAA CGG AAG ATA TAC AGC AAC TC-3') or TCR Vβ11 (5'-TCC ACA GAG AAG GGA GAT CTT TCC TCT GAG-3') region, and 1 μmol/l reverse primer specific for TCR constant α (5'-ATC ATA AAT TCG GGT AGG ATC C-3') or constant β (5'-CTC TTG ACC ATG GCC ATC-3') region. PCR was performed for 40 cycles (95°C for 20 s, 55°C for 20 s, and 72°C for 40 s) on a GeneAmp PCR system 9600 thermal cycler (Perkin Elmer, Zaventem, Belgium). PCR amplicons were used in a second amplification procedure of 25 cycles using the TCR Vα24 or TCR Vβ11 specific primer as forward primer and a FAM labelled TCR constant α (5'-FAM-CTG TTG CTC TTG AAG TCC ATA G-3') or TCR constant β (5'-FAM-GTG GCA AGG CAC ACC AGT GTG GGC C-3') as reverse primer (Eurogentec, Liege, Belgium) under the same PCR conditions as described above.

**Figure 1:**
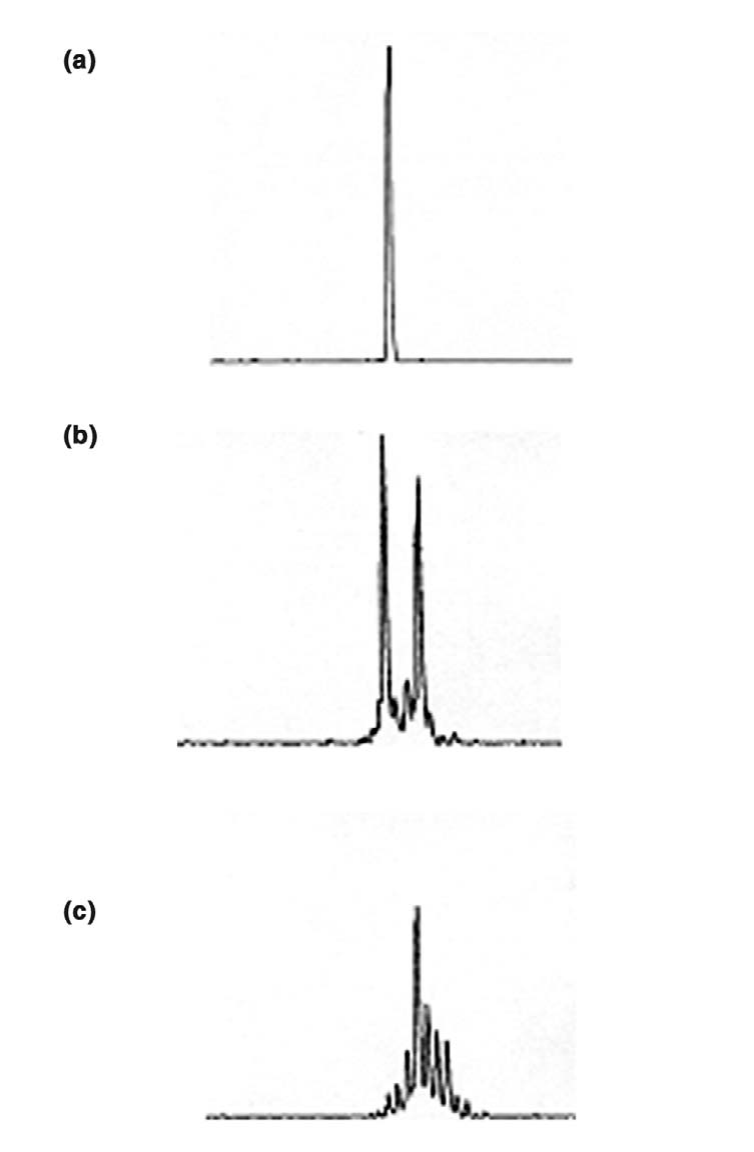
Clonality of T-cell populations. **(a) **Monoclonal: one peak. **(b) **Oligoclonal: two to four peaks. **(c) **Polyclonal: more than four peaks.

PCR amplicon lengths were analyzed on the 310 ABI DNA sequencer (Applied Biosystems, Warrington, UK). Fragment sizes of gene products were calculated using an internal Genescan-500 ROX labelled standard and analysis was performed with 672 Genescan Software (both from Applied Biosystems). The heterogeneity of the CDR3 spectratype profiles provides an indication of the clonality of T-cell populations (Fig. 1): monoclonal with one peak, oligoclonal with two to four peaks, and polyclonal with more than four peaks. Identical peak lengths strongly indicate the presence of identical T cell clones in different samples. A 350 base pair fragment was obtained for the invariant TCR.

### Sequence analysis of the invariant T-cell receptor

Purified TCR Vα24 PCR amplicons obtained from first round PCR (as described above) were sequenced with a TCR constant α primer (5'-CTG TTG CTC TTG AAG TCC ATA G-3') using the Big DyeTM Terminator Cycle Sequence Ready Reaction Kit II (Applied Biosystems). Sequences were analyzed on a ABI Prism 310 Genetic Analyser (Applied Biosystems).

### Statistical analysis

Differences in the percentage of NKT cells between healthy control individuals and RA patients and between peripheral blood and synovial fluid from RA patients were analyzed using the Mann–Whitney U-test. For comparisons between matched peripheral blood and synovial fluid samples, the Wilcoxon matched pairs signed rank test was used. *P *< 0.05 was considered statistically significant.

## Results

### Frequency of Vα24^+^Vβ11^+^CD3^+ ^natural killer T cells in rheumatoid arthritis

**Figure 2:**
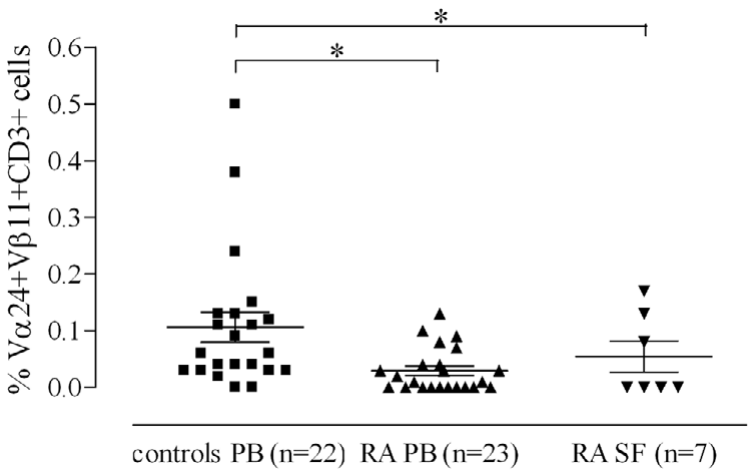
Frequency of natural killer T (NKT) cells in rheumatoid arthritis (RA) patients and healthy control individuals. NKT cell frequency in freshly isolated peripheral blood (PB) mononuclear cells from 22 healthy control individuals and 23 RA patients, and in synovial fluid (SF) mononuclear cells from seven RA patients was determined by flow cytometry. Cells were stained with anti-Vα24, anti-Vβ11 and anti-CD3 monoclonal antibody. Error bars indicate the standard error of the mean. *P < 0.01.

The frequency of Vα24^+^Vβ11^+^CD3^+ ^NKT cells in PBMCs from RA patients and healthy control individuals was analyzed by flow cytometry (Fig. 2). Significantly fewer Vα24^+^Vβ11^+^CD3^+ ^NKT cells were found in PBMCs from RA patients (0.03 ± 0.01%) than in healthy control individuals (0.11 ± 0.03%; *P *< 0.01). We simultaneously determined the NKT cell frequency in paired blood–synovial fluid samples from seven RA patients. Although a tendency toward a higher frequency was observed in the synovial fluid (0.08 ± 0.03%) as compared with the concordant PBMC samples (0.05 ± 0.02%), this finding could not be demonstrated for all patients. These data indicate that the NKT cell frequency is decreased in the blood of RA patients but not increased in synovial fluid as compared with blood from these patients.

### Cytokine profile of α-galactosylceramide stimulated peripheral blood mononuclear cells

**Figure 3:**
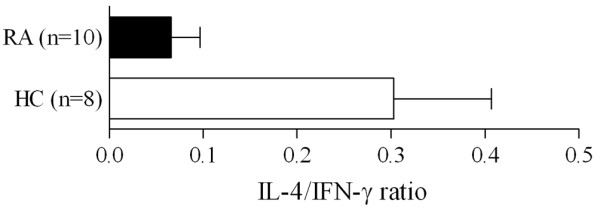
IL-4/IFN-γ ratio in α-galactosylceramide (α-GalCer) stimulated peripheral blood mononuclear cells (PBMCs) evaluated by ELISPOT. PBMCs (2 × 10^5 ^cells/well) from 10 rheumatoid arthritis patients and eight healthy control individuals were stimulated with α-GalCer or no antigen for 20 hours. The number of cytokine secreting cells was calculated by subtracting the number of spots in control wells (without antigen) from the number of spots obtained in the presence of each stimulating agent. The IL-4/IFN-γ ratio is the number of IL-4 producing cells divided by the number of IFN-γ producing cells. Error bars indicate standard error of the mean.

To assess the cytokine profile of NKT cells directly *ex vivo*, we tested the reactivity of PBMCs to α-GalCer in 10 RA patients and eight healthy control individuals using an ELISPOT technique with IFN-γ and IL-4 readout. Similar to the frequency analysis by flow cytometry, a significantly decreased number of α-GalCer reactive cells was found for IFN-γ as well as for IL-4 in RA patients as compared with healthy control individuals (2.3 ± 0.6 spots versus 24.3 ± 10.1 spots for IFN-γ and 0.2 ± 0.1 spots versus 3.9 ± 1.1 spots for IL-4 per 2 × 10^5 ^cells for RA patients and healthy control individuals, respectively; *P *< 0.05). To determine whether this diminished frequency was also associated with an altered cytokine profile, the IL-4/IFN-γ ratio was calculated as the number of IL-4 producing cells to the number of IFN-γ producing cells (Fig. 3). The IL-4/IFN-γ ratio in RA patients was decreased as compared with that in healthy control individuals (0.07 ± 0.03 in RA patients versus 0.30 ± 0.10 in healthy control individuals; *P *= 0.06). This was mainly due to a reduced number of IL-4 producing cells, because the frequency of IL-4 producing cells in RA patients as compared with healthy control individuals was relatively more reduced than that of IFN-γ producing cells. These data indicate that NKT cells derived from RA patients are biased toward a Th1-like phenotype.

### Analysis of the invariant T-cell receptor in synovial tissue

NKT cells express the invariant Vα24Jα18 TCR-α chain combined with a variable Vβ11 TCR-β chain. To compare the Vα24 expression profile in PBMCs from RA patients and healthy control individuals, PBMCs from five healthy control individuals and paired PBMCs–SFMCs and PBMCs–synovial tissue samples from four RA patients were subjected to TCR CDR3 size analysis using primers for Vα24 and TCR-α constant region. PBMCs from healthy control individuals exhibited a polyclonal peak profile or a Gaussian-like distribution for Vα24, containing a peak at 350 base pairs, which corresponds to the invariant TCR-α chain that is characteristic for NKT cells (not shown). Although PBMCs from RA patients exhibited an oligoclonal or monoclonal distribution, indicating a restricted usage for Vα24 (Table 2), the invariant TCR peak was present in all patients. We determined whether the invariant TCR could also be found in SFMCs and synovial tissue samples. As in PBMCs, the TCR Vα24 usage in SFMCs and synovial tissue tissue samples was skewed for some patients but polyclonal for others. Again, the invariant TCR peak was detected in SFMCs and synovial tissue samples for all RA patients. Sequence analysis of the PCR products obtained from the CDR3 fragment length analysis confirmed that the peak size of the synovial tissue samples corresponded with the invariant TCR sequence (not shown). These data show that NKT cells are present in rheumatoid synovial fluid as well as in synovial tissue.

### Natural killer T-cell reactivity to α-galactosylceramide in rheumatoid arthritis patients

**Figure 4:**
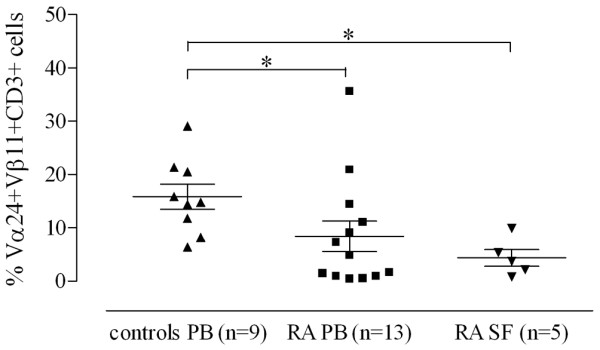
Reactivity of peripheral blood (PB) and synovial fluid (SF) derived natural killer T (NKT) cells to α-galactosylceramide (α-GalCer). PB mononuclear cells (1.5 × 10^6 ^cells/well) of nine healthy control individuals and 13 rheumatoid arthritis (RA) patients as well as SF mononuclear cells of five RA patients were stimulated with α-GalCer and re-stimulated on day 7 with autologous, α-GalCer pulsed, irradiated PB mononuclear cells in the presence of 2 U/ml IL-2. NKT cell numbers were determined by flow cytometry at day 14. Error bars indicate standard error of the mean. *P < 0.01.

**Figure 5:**
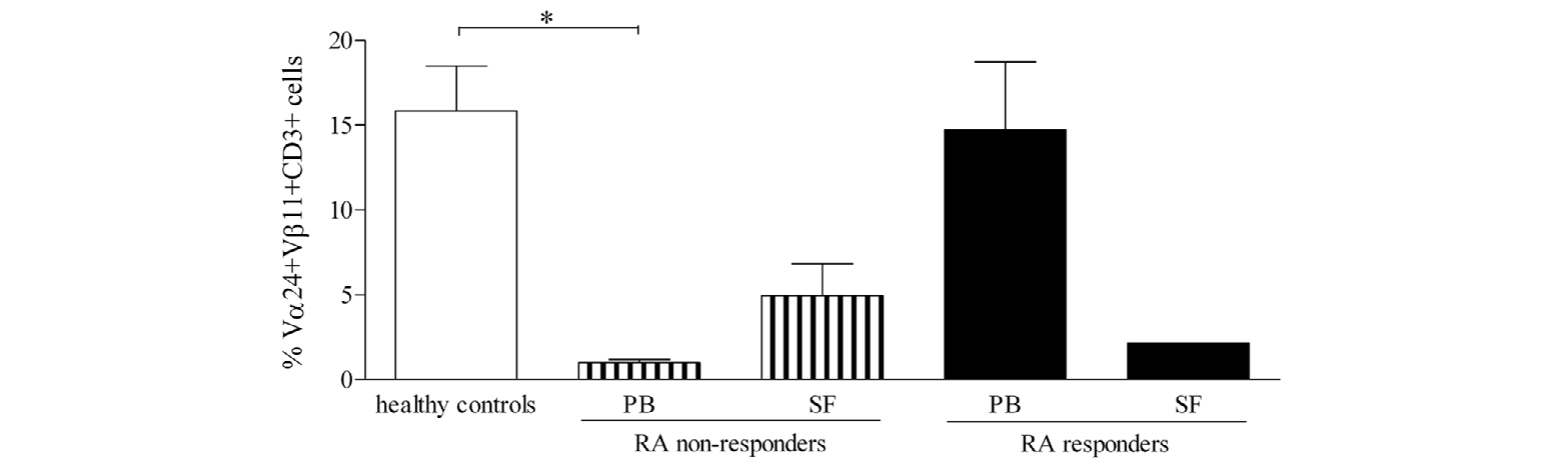
Rheumatoid arthritis (RA) patients can be divided into responder and nonresponder patients, based on peripheral blood derived natural killer T (NKT) cell reactivity to α-galactosylceramide (α-GalCer). Peripheral blood (PB) mononuclear cells (1.5 × 10^6 ^cells/well) from nine healthy control individuals and 13 RA patients, as well as synovial fluid (SF) mononuclear cells from five RA patients, were stimulated with α-GalCer and re-stimulated on day 7 with autologous, α-GalCer pulsed, irradiated PB mononuclear cells in the presence of 2 U/ml IL-2. NKT cell numbers were determined by flow cytometry on day 14. Patients were considered nonresponders when the frequency of Vα24^+^Vβ11^+^CD3^+ ^NKT cells derived from PB mononuclear cells was lower than 2% after 14 days of culture. Error bars indicate standard error of the mean. *P < 0.01.

To assess whether the reduced NKT cell frequency in peripheral blood from RA patients was due to an inadequate response to the glycolipid antigen, we stimulated PBMCs from nine healthy control individuals and 13 RA patients and SFMCs from five RA patients with α-GalCer. At day 7, cells were re-stimulated with autologous α-GalCer pulsed, irradiated PBMCs. The NKT cell frequency was determined by flow cytometry at day 14 (Fig. 4). NKT cells from healthy control individuals expanded in response to α-GalCer to 15.8 ± 2.7%, whereas the number of peripheral blood and synovial fluid NKT cells from RA patients was significantly lower after α-GalCer stimulation (8.4 ± 2.9% and 4.4 ± 1.6%, respectively; *P *< 0.01). A more detailed analysis revealed that this decrease was due to the existence of two subpopulations of RA patients based on the NKT cell numbers reached after 14 days of α-GalCer stimulation. As shown in Fig. 5, NKT cells from six out 13 RA patients did not respond to α-GalCer stimulation (mean frequency after 14 days: 1.0 ± 0.2%, *P *< 0.01; nonresponders), whereas NKT cells from the remaining seven patients reached frequencies comparable with those in healthy control individuals (14.7 ± 4.0%; responders). Moreover, NKT cells of responder patients appeared to have increased ability to respond to α-GalCer because the expansion was greater than that in healthy control individuals (294-fold versus 149-fold, respectively). No relation between disease parameters (disease duration, disease status) or treatment and responsiveness/nonresponsiveness of NKT cells could be demonstrated. Remarkably, synovial fluid NKT cells, even from nonresponding RA patients, did expand after α-GalCer stimulation (4.94 ± 1.90%). These findings indicate that the reactivity of peripheral blood NKT cells to α-GalCer is impaired in some RA patients, whereas it is intact and even increased in others.

### Cytokine profile of peripheral blood and synovial fluid natural killer T cell lines

**Figure 6:**
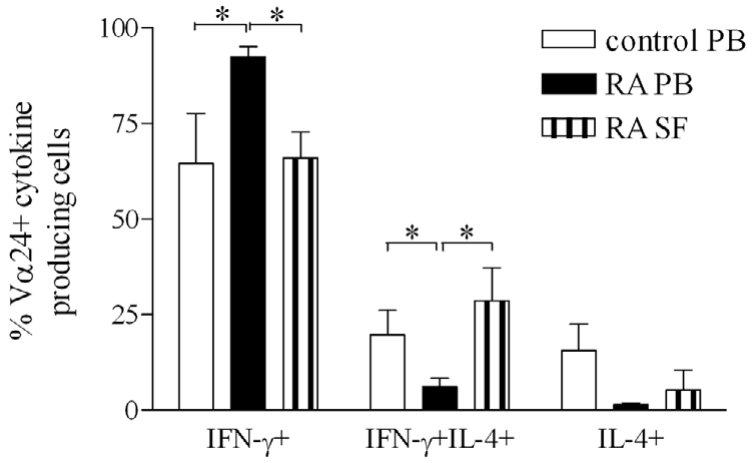
Cytokine profile of α-galactosylceramide (α-GalCer) expanded natural killer T (NKT) cells. Peripheral blood (PB) mononuclear cells (1.5 × 10^6 ^cells/well) from five healthy control individuals and five RA patients as well as synovial fluid (SF) mononuclear cells from five RA patients were stimulated with α-GalCer and re-stimulated on day 7 with autologous, α-GalCer pulsed, irradiated PB mononuclear cells in the presence of 2 U/ml IL-2. The cytokine profile was analyzed by intracellular staining and gating on the Vα24^+ ^subset. Error bars indicate standard error of the mean. *P < 0.05.

Next, we analyzed the cytokine profile of peripheral blood derived NKT cells from five healthy control individual and five RA patients, and synovial fluid derived NKT cells from five RA patients by intracellular staining of 14-day-old, α-GalCer stimulated cultures gated on Vα24^+ ^cells. Figure 6 shows that the Vα24^+ ^NKT cell fraction of healthy control individuals contained 64.5 ± 13.1% IFN-γ producing cells, 15.7 ± 6.9% IL-4 producing cells, and 19.7 ± 6.4% cells producing both IFN-γ and IL-4. In contrast, peripheral blood NKT cells from RA patients consisted of significantly more IFN-γ producing cells and significantly fewer cells producing both IFN-γ and IL-4 (92.5 ± 2.7% and 6.1 ± 2.3%, respectively; *P *< 0.05). Remarkably, synovial fluid derived NKT cells exhibited a cytokine profile similar to that of healthy control individuals, although the number of IL-4 producing cells tended to be lower and the number of cells producing both IFN-γ and IL-4 was somewhat higher (5.3 ± 5.3% and 28.7 ± 6.7%, respectively; *P *> 0.05). No differences were found between the cytokine profiles of NKT cells of α-GalCer responding and nonresponding patients. Furthermore, no relation with treatment or any disease parameter was found. These observations show that, although NKT cells in PBMCs from RA patients are biased toward a Th1-like cytokine profile, NKT cells in the synovial fluid exhibit a Th0-like cytokine profile that is comparable with that in healthy control individuals.

### Cytokine profile of CD4^+ ^and CD4^- ^natural killer T cell subsets in patients with rheumatoid arthritis and healthy control individuals

The observed Th1-like bias in NKT cells from RA patients might be due to an increased number of double-negative NKT cells or a decreased number of CD4^+ ^NKT cells. To analyze the frequency of these NKT cell subtypes, we isolated the Vα24^+ ^cells of α-GalCer stimulated, 14-day-old cultures derived from PBMCs from nine healthy control individuals and seven RA patients by immunomagnetic selection. Positively selected cells were tested for α-GalCer reactivity to ensure the NKT cell nature of the cells. The presence of CD4 was assessed by flow cytometry. NKT cells of healthy control individuals consisted of 33.3 ± 6.7% CD4^+ ^NKT cells and 66.7 ± 6.7% CD4^- ^(double-negative) NKT cells. The frequency of CD4^+ ^and CD4^- ^NKT cells in RA patients did not differ significantly from that in healthy control individuals (49.8 ± 6.3% and 50.2 ± 6.3%, respectively; data not shown).

**Figure 7:**
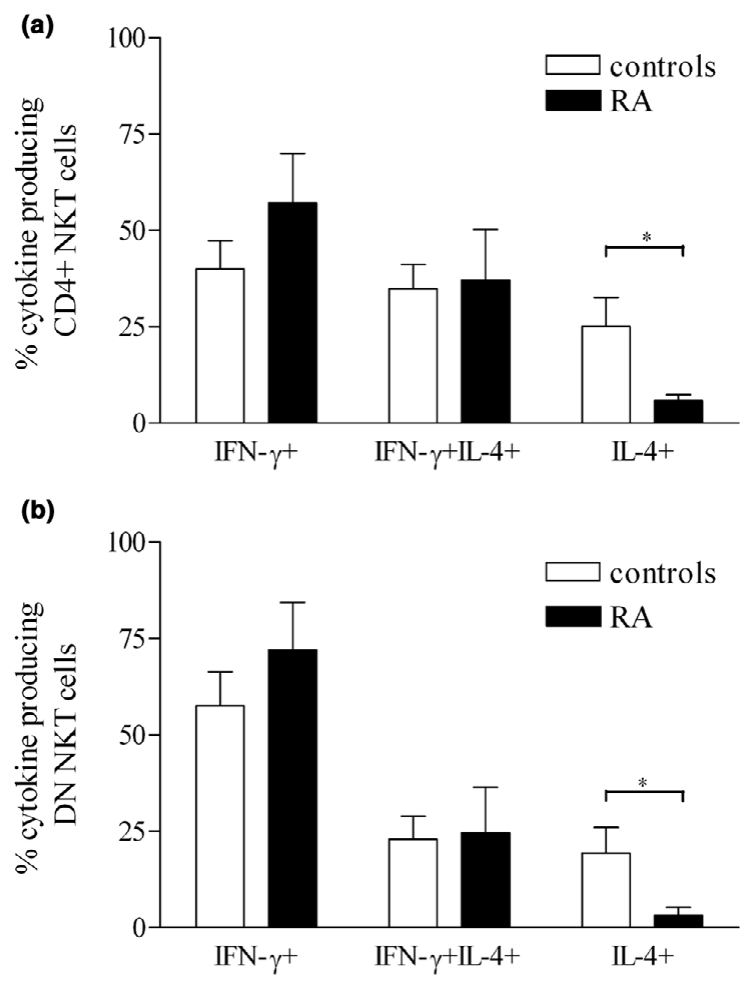
Cytokine profile of CD4^+ ^and CD4^- ^natural killer T (NKT) cell lines derived from peripheral blood mononuclear cells from rheumatoid arthritis (RA) patients and healthy control individuals. Vα24^+ ^cells of α-galactosylceramide (α-GalCer) stimulated, 14-day-old cultures from nine healthy control individuals and nine RA patients were isolated using biomagnetic selection. The cytokine profile of **(a) **CD4^+ ^and **(b) **CD4^- ^NKT cells was assessed by intracellular staining. Error bars indicate standard error of the mean. *P < 0.05.

Figure 7 shows the cytokine profile of each NKT cell subset, as determined by intracellular staining. Peripheral blood derived CD4^- ^NKT cells from healthy control individuals predominantly consisted of IFN-γ producing cells (IFN-γ^+ ^57.6 ± 8.8%; IL-4^+ ^19.4 ± 6.6%; IFN-γ^+^IL-4^+ ^23.0 ± 6.0%), whereas CD4^+ ^NKT cells contained almost as many IL-4 producing cells as IFN-γ producing cells (IFN-γ^+ ^40.1 ± 7.4%; IL-4^+ ^25.1 ± 7.5%; IFN-γ^+^IL-4^+ ^34.8 ± 6.4%). However, the CD4^+ ^as well as the CD4^- ^NKT cell fractions in RA patients contained significantly fewer IL-4 producing cells as compared with their counterparts in healthy control individuals (for CD4^+ ^NKT cells: IFN-γ^+ ^57.2 ± 12.9%; IL-4^+ ^5.8 ± 1.5%; IFN-γ^+^IL-4^+ ^37.0 ± 13.2%; and for CD4^- ^NKT cells: IFN-γ^+ ^72.1 ± 12.4%; IL-4^+ ^3.3 ± 1.9%; IFN-γ^+^IL-4^+ ^24.6 ± 11.9%), indicating that both CD4^+ ^and CD4^- ^NKT cells in the peripheral blood of RA patients are biased toward a Th1-like cytokine profile.

To exclude the possibility that the observations in NKT cell lines of RA patients were caused by the clonal expansion of one or a few NKT cells, we analyzed the heterogeneity of the Vα24 and Vβ11 TCR by means of CDR3 fragment length analysis. We found that the NKT cell lines of both RA patients and healthy control individuals exhibited a monoclonal Vα24 and polyclonal Vβ11 profile (data not shown), which shows that the differences between NKT cells from RA patients and healthy control individuals found in response to α-GalCer are not due to a skewed outgrowth of only one or a few NKT cells.

## Discussion

Several studies have provided evidence that NKT cells are involved in autoimmune conditions [27]. Attempts to increase the number of NKT cells in animal models of autoimmunity by transgenic expression of the invariant TCR or by passive transfer of NKT cells resulted in a protective effect against disease induction [28,29]. Additionally, administration of α-GalCer resulted in prevention or suppression of disease. These studies indicate that NKT cells can play a role in the regulation of autoimmunity and that they are therefore an interesting subject for further investigation in human autoimmune diseases.

In the present study we demonstrated a decreased frequency of NKT cells in PBMCs from RA patients. Because we used anti-Vα24 and anti-Vβ11 monoclonal antibodies to identify invariant NKT cells, it is possible that conventional T cells were also stained by this combination. However, Araki and coworkers [12] showed that the frequency of Vα24^+^Vβ11^+^CD3^+ ^T cells, even at low numbers, corresponded well with the NKT cell frequency determined by CD1d tetramers, which supports the specificity of anti-Vα24 and anti-Vβ11 staining for NKT cells.

Several mechanisms may account for NKT cell reduction in the peripheral blood of RA patients. First, NKT cells might preferentially migrate into the joint to fulfill their regulatory function. We therefore studied the frequency of NKT cells in synovial fluid and synovial tissue of RA patients. We found that the NKT cell frequency is not elevated in synovial fluid, but that the invariant TCR can be detected in both synovial tissue and synovial fluid samples from RA patients. Preferential migration of NKT cells into the synovium may have resulted in a monoclonal or oligoclonal Vα24 profile in synovial samples. However, we did not find such a profile in the synovial fluid or synovial tissue of all patients, indicating that the decrease cannot be accounted for by a selective migration of NKT cells toward the joint. A similar conclusion was reached by others for RA [30] and multiple sclerosis [31].

A second possibility might be that the reduced NKT cell frequency is caused by a selective loss of a limited number of NKT cell clones. It was shown in mice that NKT cells exhibit a highly diverse TCR-β repertoire and a small clone size [32], and hence a loss of NKT cells should result in a reduced diversity of TCR Vβ11. However, the Vβ11 profile of α-GalCer expanded peripheral blood NKT cells from RA patients was polyclonal, which suggests that RA patients do not suffer from a specific loss of NKT cells.

A third possible cause is a decreased reactivity toward the natural NKT cell ligand. To examine this possibility, we stimulated PBMCs of RA patients with α-GalCer and found that, in 53.8% of the patients ('responders'), NKT cells expanded upon α-GalCer stimulation and reached levels comparable to those in healthy control individuals. This suggests that an inadequate expression of CD1d [33] or an aberrant presentation of the natural NKT cell antigen, but not decreased reactivity, might account for the NKT cell reduction in these responder patients. In contrast, in 46.2% of the patients ('nonresponders') NKT cells did not react to α-GalCer. This impaired NKT cell function was also reported previously by Kojo and coworkers [11], who proposed that this decreased reactivity might result from an inherent NKT cell defect or a dysfunctional antigen presentation. However, those authors could exclude the possibility that antigen-presenting cells were dysfunctional in nonresponder patients. Remarkably, synovial fluid NKT cells of both responders and nonresponders expanded upon stimulation, indicating that the impaired NKT cell function in nonresponders is restricted to the blood compartment.

Additional mechanisms may account for the reduced frequency, including a decreased thymic output, as was described previously for conventional T cells in RA [34], and a chronic over-stimulation of NKT cells resulting in a decreased frequency due to TCR downregulation after activation [35]. Moreover, it is possible that a chronic activation might also lead to nonresponsiveness because it was shown that NKT cells in α-GalCer injected mice are anergic for an extended period of time [36].

When we analyzed the cytokine profiles of *in vitro *expanded NKT cells, we found that CD4^- ^NKT cells from healthy control individuals mainly consisted of IFN-γ producing cells, whereas CD4^+ ^NKT cells can produce both Th1-like and Th2-like cytokines. This reflects the direct *ex vivo *situation reported by others [2,3]. We observed that peripheral blood derived NKT cells from RA patients exhibited a Th1-like phenotype, which was due to a decreased number of IL-4 producing cells in both the CD4^+ ^and CD4^- ^NKT cell subsets compared with healthy control individuals. Although these data were obtained from *in vitro *cultured cells, our data obtained from direct *ex vivo *stimulation of PBMCs with α-GalCer confirm a Th1-like bias of NKT cells in RA patients. Strikingly, NKT cells in the synovial fluid do not show this Th1-like bias, but have a Th0-like profile that is similar to that of peripheral blood NKT cells from healthy control individuals. A Th1-like bias of peripheral blood derived NKT cells was also found in diabetes [9] and multiple sclerosis [12], indicating that NKT cell dysfunction is not specific for RA but might play a major role in the aetiology of autoimmune diseases.

Although no relation between reactivity to α-GalCer or NKT cell cytokine profiles and drug treatment was found, a possible effect of the medication cannot be excluded.

In summary, the presence, even in nonresponder patients, of functional NKT cells that exhibit a Th0-like cytokine profile in the synovial fluid may indicate that unimpaired NKT cells migrate from the peripheral blood toward the synovium in order to exert their regulatory function. NKT cells express a chemokine receptor profile similar to Th1-type inflammatory homing cells, which suggests that these cells perform their function mainly in the tissue [37]. However, their number and/or function are probably insufficient to resolve the ongoing autoimmune reaction. Hence, a strategy to enhance locally the number of NKT cells by α-GalCer represents a potential treatment for RA.

## Conclusion

Because the number of Vα24^+^Vβ11^+^CD3^+ ^NKT cells is decreased and the cytokine profile of blood derived NKT cells is biased toward a Th1-like phenotype in RA patients, NKT cells might be functionally related to resistance or progression of RA and are therefore an interesting target for the treatment of RA.

## Abbreviations

α-GalCer = α-galactosylceramide; FITC = fluorescein isothiocyanate; IFN = interferon; IL = interleukin; NKT = natural killer T (cell); PBMC = peripheral blood mononuclear cell; PE = phycoerythrin; PCR = polymerase chain reaction; RA = rheumatoid arthritis; SFMC = synovial fluid mononuclear cell; TCR = T-cell receptor; Th = T-helper (cell).

## Competing interests

The author(s) declare that they have no competing interests.

## Authors' contributions

LL carried out all experiments and drafted the manuscript. MT participated in frequency analysis of NKT cells. KB participated in reactivity assays. PG provided clinical material. VS and JR critically revised the manuscript. PS coordinated the study. All authors read and approved the final manuscript.

## Figures and Tables

**Table 1:** Patient characteristics

Patient	Age (years)/sex	Disease duration (years)	Treatment
1	54/M	5	Azathioprine, methylprednisolone
2	38/F	6	Hydroxychloroquine, salazopyrine
3	64/F	7	NSAID
4	43/F	5	NSAID
5	46/M	1	Salazopyrine
6	46/M	<1	Salazopyrine
7	52/M	11	NSAID
8	53/F	11	NSAID
9	49/M	10	NSAID
10	52/F	4	NSAID
11	69/F	36	Salazopyrine
12	65/F	<1	Untreated
13	35/M	<1	Untreated
14^a^	57/F	4	Methotrexate
15^a^	46/M	2	Anti-TNF, salazopyrine
16^a^	41/M	10	Salazopyrine
17^a^	41/M	13	Methotrexate
18^a^	60/M	2	Leflunomide
19^a^	43/F	5	Methotrexate
20^b^	63/M	17	Salazopyrine
21^a,b^	65/F	4	Salazopyrine, hydroxychloroquine
22^b^	62/F	12	Leflunomide
23^b^	54/F	17	Methylprednisolone

^a^Synovial fluid sample. ^b^Synovial tissue sample. F, female; M, male; NSAID, nonsteroidal anti-inflammatory drug; TNF, tumour necrosis factor.

**Table 2:** T cell receptor Vα24 usage in peripheral blood mononuclear cells, synovial fluid mononuclear cells and synovial tissue from rheumatoid arthritis patients

	Vα24			Vβ11		
	
	PBMCs	SFMCs	ST	PBMCs	SFMCs	ST
	
RA 1	mono	mono	NA	oligo (2)	mono	NA
RA 2	mono	poly	oligo (2)	mono	poly	oligo (2)
RA 3	mono	oligo (2)	NA	Poly	oligo (2)	NA
RA 4	poly	poly	NA	Poly	poly	NA
RA 5	oligo (3)	NA	mono	mono	NA	mono
RA 6	poly	NA	poly	poly	NA	poly
RA 7	oligo (3)	NA	mono	poly	NA	oligo (3)

The clonality of the T-cell receptor (TCR) Vα24 family was assessed by CDR3 spectratyping of peripheral blood mononuclear cells (PBMCs), synovial fluid mononuclear cells (SFMCs) and synovial tissue (ST) from rheumatoid arthritis (RA) patients. (See Fig. 1 for representative monoclonal [panel a], oligoclonal [panel b] and polyclonal [panel c] profiles.) mono, monoclonal profile; NA, not available; oligo, oligoclonal profile; poly, polyclonal profile.
